# Brain-computer interface combined with mental practice and occupational therapy enhances upper limb motor recovery, activities of daily living, and participation in subacute stroke

**DOI:** 10.3389/fneur.2022.1041978

**Published:** 2023-01-09

**Authors:** Aristela de Freitas Zanona, Daniele Piscitelli, Valquiria Martins Seixas, Kelly Regina Dias da Silva Scipioni, Marina Siqueira Campos Bastos, Leticia Caroline Kaspchak de Sá, Kátia Monte-Silva, Miburge Bolivar, Stanislaw Solnik, Raphael Fabricio De Souza

**Affiliations:** ^1^Department of Occupational Therapy and Graduate Program in Applied Health Sciences, Federal University of Sergipe, São Cristóvão, Sergipe, Brazil; ^2^School of Medicine and Surgery, University of Milano-Bicocca, Milan, Italy; ^3^Department of Kinesiology, University of Connecticut, Storrs, CT, United States; ^4^Department of Occupational Therapy, Federal University of Paraná, Curitiba, Paraná, Brazil; ^5^Department of Physical Therapy, Federal University of Pernambuco, Recife, Pernambuco, Brazil; ^6^Department of Physical Therapy, University of North Georgia, Dahlonega, GA, United States; ^7^Department of Physical Education, Wroclaw University of Health and Sport Sciences, Wroclaw, Poland

**Keywords:** stroke, brain-computer interface, occupational therapy, mental practice, rehabilitation

## Abstract

**Background:**

We investigated the effects of brain-computer interface (BCI) combined with mental practice (MP) and occupational therapy (OT) on performance in activities of daily living (ADL) in stroke survivors.

**Methods:**

Participants were randomized into two groups: experimental (*n* = 23, BCI controlling a hand exoskeleton combined with MP and OT) and control (*n* = 21, OT). Subjects were assessed with the functional independence measure (FIM), motor activity log (MAL), amount of use (MAL-AOM), and quality of movement (MAL-QOM). The box and blocks test (BBT) and the Jebsen hand functional test (JHFT) were used for the primary outcome of performance in ADL, while the Fugl-Meyer Assessment was used for the secondary outcome. Exoskeleton activation and the degree of motor imagery (measured as event-related desynchronization) were assessed in the experimental group. For the BCI, the EEG electrodes were placed on the regions of FC3, C3, CP3, FC4, C4, and CP4, according to the international 10–20 EEG system. The exoskeleton was placed on the affected hand. MP was based on functional tasks. OT consisted of ADL training, muscle mobilization, reaching tasks, manipulation and prehension, mirror therapy, and high-frequency therapeutic vibration. The protocol lasted 1 h, five times a week, for 2 weeks.

**Results:**

There was a difference between baseline and post-intervention analysis for the experimental group in all evaluations: FIM (*p* = 0.001, *d* = 0.56), MAL-AOM (*p* = 0.001, *d* = 0.83), MAL-QOM (*p* = 0.006, *d* = 0.84), BBT (*p* = 0.004, *d* = 0.40), and JHFT (*p* = 0.001, *d* = 0.45). Within the experimental group, post-intervention improvements were detected in the degree of motor imagery (*p* < 0.001) and the amount of exoskeleton activations (*p* < 0.001). For the control group, differences were detected for MAL-AOM (*p* = 0.001, *d* = 0.72), MAL-QOM (*p* = 0.013, *d* = 0.50), and BBT (*p* = 0.005, *d* = 0.23). Notably, the effect sizes were larger for the experimental group. No differences were detected between groups at post-intervention.

**Conclusion:**

BCI combined with MP and OT is a promising tool for promoting sensorimotor recovery of the upper limb and functional independence in subacute post-stroke survivors.

## Introduction

Epidemiological data suggest that about one-third of the 16 million/year patients with post-stroke worldwide remain with significant limitations in engaging in meaningful activities of daily living (ADL) and performing tasks with satisfactory upper limb (UL) function (1).

The inability to use the affected UL after injury may be related to sensory and motor brain impairments due to decreased cortical excitability and an imbalance of interhemispheric competition (2–5). Thus, elucidating the mechanisms that will optimize neuroplasticity during rehabilitation treatment, increase cortico-cerebral excitability, and facilitate long-term functional recovery becomes a significant challenge (6).

Artificial intelligence and neuroengineering technologies such as the brain-computer interface (BCI) can promote improved brain plasticity and functional reorganization of the brain, which is a promising approach for post-stroke rehabilitation, especially to improve arm motor function (6).

In its entirety, BCI is an innovative intervention that decodes neural signals by electroencephalogram (EEG) in real-time, transferring to digital signals that activate a device, prostheses, or robots, triggering and providing instant multimodal feedback (visual, sensory, and kinesthetic) to the coupled member (7). In EEG-based non-invasive BCI, movement intention or mental practice (MP) is decoded in real-time from the ongoing electrical activity of the brain (6). The EEG can analyze related brain waves in the premovement period: Bereitschaftspotential (which can be recorded over the vertex region), and during the movement tasks, mu and beta rhythms were found to reveal event-related synchronization and desynchronization (ERS/ERD) over the sensorimotor cortex (8).

Notably, studies showed that BCI was able to promote increased activation of the primary (M1) and frontal motor cortex, thus promoting a process of brain reorganization and neuroplasticity (1, 7, 9, 10).

Recent studies have shown the effectiveness of applying BCI using mental practice in motor recovery for subacute and chronic stroke patients with hemiparesis (6, 11–19). Furthermore, a relevant rehabilitation study combing BCI and mental practice showed promising results for the recovery of cognitive skills (executive functions, language, memory, attention, and visuospatial skills) in post-stroke patients (20).

As demonstrated, it is understandable to perceive the potential of BCI as a supporting strategy for improving brain plasticity and UL motor functions after stroke. However, knowing that the improvement of a skill *per se* may not reflect the improvement of functional independence for activities of daily living, social participation, and occupational performance. The World Health Organization's (WHO) International Classification of Functioning, Disability and Health (ICF), describes the activity as the performance of a task or action by an individual and participation as its involvement in real situations of daily life (21).

Thus, this study aimed to investigate the effects of BCI combined with MP and occupational therapy (OT) on the manual function to improve performance in executing ADL and increase the social participation of stroke survivors in the subacute phase.

## Materials and methods

### Study design

A randomized clinical trial, characterized by double blinding (evaluator and statistician) was conducted. The study was approved by the Ethics Committee in Research with Human Beings of the Health Sciences Center, Federal University of Sergipe, Brazil (65123016.5.0000.5546). The present study followed the CONSORT (Consolidated Standards of Reporting Trials) guidelines.

The study was carried out at the Laboratory of Studies in Neurological Learning and Rehabilitation (LEARN) at the University of the Federal University of Sergipe, located in Lagarto, from August 2019 to May 2022.

Once the participants were recruited, an initial screening was performed to determine the eligibility criteria. For participants included, a battery of assessments was performed using the instruments for the primary and secondary outcomes. After being evaluated, the participants were randomized and allocated to the trial groups.

#### Experimental group

BCI; mental practice with functional tasks; and OT with training in ADL, muscle mobilization, reaching tasks, manipulation and grip, mirror therapy, and high-frequency therapeutic vibration. The protocol lasted 80 min, five times a week, totaling ten intervention sessions.

#### Control group

A protocol was carried out only with the occupational therapy intervention, which included training in ADL, muscle mobilization, reaching tasks, manipulation and grip, mirror therapy, and high-frequency therapeutic vibration. Patients in the control group did not do imagery tasks. Only the occupational therapy protocol was used (no mental practice with functional tasks or BCI).

The protocol lasted 80 min, five times a week, totaling ten intervention sessions.

### Population

Individuals were recruited through social media and referred by local hospitals, rehabilitation centers, and health centers. The sample consisted of individuals with a confirmed diagnosis of ischemic or hemorrhagic stroke, who had been affected by a single episode in the early subacute stage (from 3 weeks to 3 months of stroke) and late subacute stage (from 3 to 6 months after the stroke) (22), who were between 35 and 80 years of age with motor impairment in the UL, and who had partially preserved cognitive function.

Individuals who had partially preserved motor and sensory function according to the Fugl-Meyer assessment scale (with scores between 10 and 60 in the motor domain and 2 and 10 in the sensory domain) in the UL contralateral to the lesion and who were partially cognitive were included in the study (cut-off point of 18 in the Mini-Mental State Examination). Due to the need for at least partially preserved cognitive ability so that the participant could perform the movement imagination, we decided to exclude those with severe cognitive deficits. We defined no deficit as scores between 24 and 30, mild impairment as scores between 18 and 24, and severe impairment as scores between 0 and 17 (23, 24).

Exclusion criteria included: individuals who underwent external rehabilitation treatments with multiple brain injuries or other neurological diseases or musculoskeletal and psychiatric disorders, individuals who had a history of seizures, individuals with UL amputations, individuals undergoing decompressive craniectomy or with metallic implants in the head, and individuals who did not sign the free and informed consent form.

#### Randomization and blinding

The participants were randomized and allocated to two groups of equal size. A stratified block allocation based on stroke onset and age was generated at www.randomization.com by an independent researcher and packaged into sequentially numbered, opaquely sealed envelopes. A researcher who did not participate in the evaluations or interventions generated the random allocation sequence, enrolled participants, and assigned participants to the interventions. Those evaluating and analyzing the outcomes and participants were blinded to the treatment arm.

### Intervention

The interventions were conducted by a qualified healthcare professional. The sessions took place in a reserved, ventilated room at the Laboratory of Studies in Neurological Learning and Rehabilitation (LEARN-UFS).

The experimental group received BCI therapy (30 min), combined with mental practice (15 min), and occupational therapy (35 min). The Occupational Therapy protocol consisted of ADL training (according to the patient's functional needs), mirror therapy; reaching, manipulating, and releasing objects tasks; muscle mobilization; and therapeutic vibration. The control group received only the occupational therapy protocol (80 min), without BCI or mental practice.

#### Brain-computer interface

The equipment used for BCI was developed by Neurobots^®^ (Exobots System Software: 1.10.0, Exobots Firmware version 2, EEG Firmware version 1). Exobots Battery: Li-Ion 3.6 V 3,000 mAh; EEG Battery: Li-Po 3.7 V 1,300 mAh. Equipment power supply voltages and frequencies: Exobots: 220 V and Freq 50/60 Hz. EEG: USB 2.0 port−12 V continuous frequency. EEG conditioning: 24 bit AD conversion resolution; sample rate: 250 Hz. Biomarkers (from EEG): alpha-band desynchronization.

**Figure 1:**
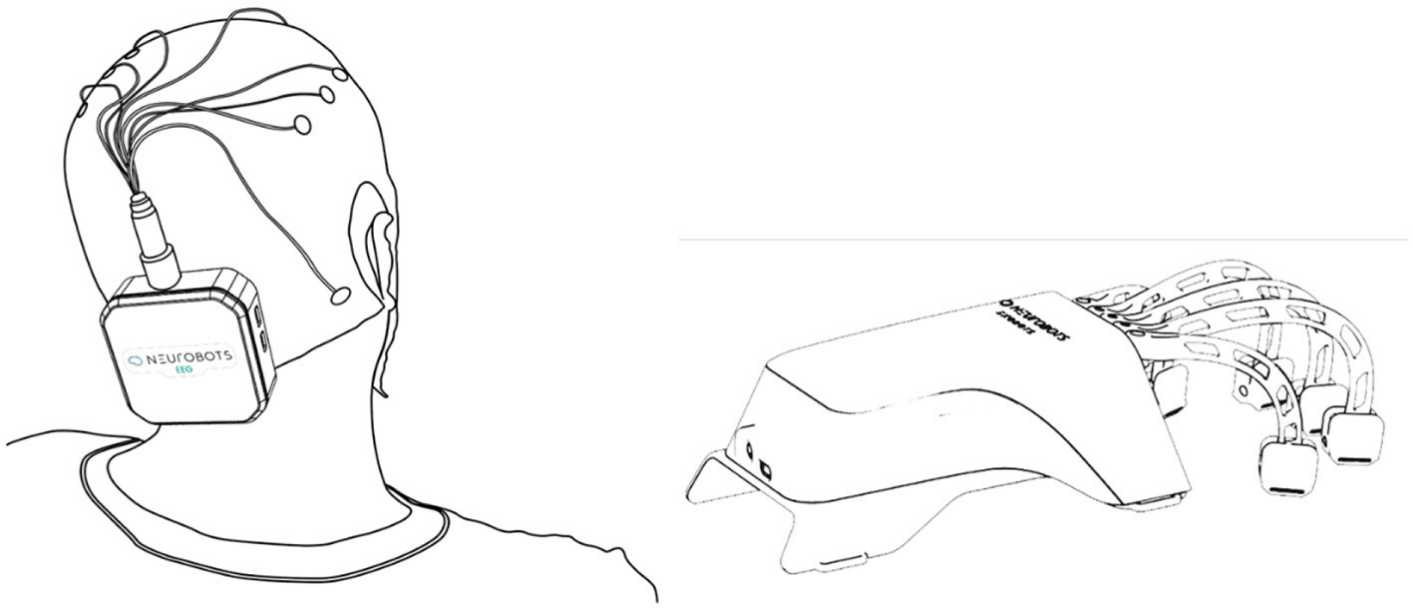
Schematic drawing of the BCI equipment.

Initially, the participant was registered in the software, and then the electroencephalogram (EEG) capture electrodes were mounted. The electrodes were placed on the regions of FC3, C3, CP3, FC4, C4, and CP4, according to the international 10–20 EEG system. FC3/FC4 lies over the premotor cortex, and C3/C4 lies over the primary motor cortex. CP3/CP4 corresponds to the supramarginal gyrus, which is part of the somatosensory association cortex. These six electrodes covered the major part of the mirror neuron system. A ground electrode was placed on the forehead, and the reference electrode was placed on either A1 or A2 in the earlobe (25–27). The affected hand rested on a pillow with the exoskeleton individually adjusted for each participant. The wrist was kept in a neutral position. A UL exoskeleton, i.e., the Exobots, was positioned over the user's wrist and fingers and fixed with five velcros that were adjusted on the fingertip and two velcros placed on the forearm. The EEG was linked to a connector consisting of six electrodes. The EEG and the electrodes were fixed to a cap placed on the participant's head (Figure 1).

During the therapy session, the individual was instructed on four auditory commands given by the software, each with a different meaning: “Relax,” “Prepare,” “Think,” and “Move.” During the “Relax” phase, the patient was instructed to remain with an empty mind without thinking and just follow the command. The software detected and showed the relaxed state quantitatively on a feedback bar. In the “Prepare” and “Think” phases, the individual was instructed to imagine performing specific movements with the most affected hand (i.e., opening or closing the hand). If the system detected a continuous activation in the primary motor, premotor, or primary somatosensory cortices above the threshold shown on the screen (i.e., 70 points) for at least 3 s, the exoskeleton opened and closed the participant's hand. Finally, in the “Move” phase, the exoskeleton automatically opened and closed the individual's hand (without motor imagery). At the end of each trial, a score representing the degree of motor imagery was shown on the screen next to the brain areas that were most activated during the session.

When performing motor imaginary (i.e., mental practice), neurons increase their activation in the motor cortex by synchronizing their action potential at a high frequency (76–100 Hz) (28). This phenomenon is known as event-related synchronization (ERS) (29). Conversely, there is a reduction in neuronal activation in motor areas at low frequencies (8–32 Hz), a phenomenon known as event-related desynchronization (ERD) (29). Interestingly, real-time feedback of cortical activation may amplify ERS at high frequencies and ERD at low frequencies, showing that BCI can potentially promote neuronal activation in cortical areas (28).

In order to capture the motor imagery through non-invasive electrical sensors, a better signal-to-noise ratio is obtained at low frequencies, thus, the software needs to identify when the user performs an ERD in the μ frequency range (8–13 Hz). To calculate the ERD, the following formula is adopted: ERD % = (*R* – *A*)/*R* × 100 (29), where R is the neuronal activation power at the μ frequency during the reference period (“Relax” phase), and A is the power of neuronal activation during motor imagery (“Think” phase). Then, the ERD is calculated on the six electrodes positioned over the motor cortex, and a degree of motor imagery is computed from the sum of the ERD of all electrodes. The degree of motor imagery was the nomenclature used by the manufacturer's BCI system, representing the ERD achieved by the user during motor imagery. During the “Think” phase, the exoskeleton was activated if there was a degree of motor imagery above an established threshold for 3 s. This time window was established to avoid false positives. The threshold was defined based on the expected ERD activity (i.e., from 50 to 100%) that occurs during motor imagery in the μ frequency range on electrodes placed over the motor cortex (29). The manufacturer defined 70% of ERD (i.e., 70 points) as the activation threshold, an intermediate value between the observed percentage ranges.

Eight electroencephalogram (EEG) electrodes attached to a cap, positioned in the region of FC3, FC4, C3, C4, CP3, CP4, ground and reference electrode of EEG system 10-10 marking. The exoskeleton is positioned over the patient's hand with articulated fingers to be fitted to the individual's fingers.

The training with the BCI consisted of 10 sets with 10 repetitions of the “Relax,” “Prepare,” “Think,” and “Move” phases. At the end of each series, a 30-s interval allowed the participant to rest and receive the results. At the end of the process, the software showed the general results of the session (i.e., degree of motor imagery and activated brain areas).

In summary, the system is controlled through the ERD value (28, 29), obtained from the imagination of the movement. Six active EEG electrodes capture the ERD value and send it to software developed by Neurobots. If the ERD value is ≥70 points and lasts for at least 3 s, a command to move the exoskeleton is sent. Thus, the exoskeleton is moved by the opening and closing of the hand in imagination. The software also automatically moves the exoskeleton after the “think” phase. This is important to offer parameters to learn the ideal movement.

#### Mental practice with functional tasks

The MP sessions were individualized, focusing on ADL training according to the individual's needs. Each ADL was divided into kinematic components, i.e., the practice of each motor component that built a whole motor task. During MP, the participant observed the movement (made by the occupational therapist), imagined each component of the task with closed eyes, and finally performed the movement, completing the functional task. Note that MP with the functional task was administered to the experimental group.

#### Occupational therapy intervention

The set of activities performed by the occupational therapist consisted of ADL training according to the patient's functional needs. Furthermore, rehabilitation interventions were administered: mirror therapy; reaching, manipulating, and releasing objects tasks; muscle mobilization; and therapeutic vibration.

For the mirror therapy protocol, the subject was positioned comfortably seated in front of a table, with both ULs forward. The affected UL was positioned behind a mirror (size 50 × 50 cm), and only the unaffected limb performed the activities. The participant was encouraged to follow all the exercises while being instructed by the occupational therapist. The following movements were performed: elbow flexion and extension, forearm pronation and supination, wrist flexion and extension, finger flexion and extension (30), and functional tasks, e.g., eating and combing hair.

Reaching, manipulating, and releasing objects were trained to favor the functional independence of individuals in their ADL. The task consisted of performing a reaching task with an everyday object positioned on the table, holding and manipulating it, moving the object in several directions around the table, simulating its functional use, and finally releasing the object.

Muscle mobilization was performed over the flexor and extensor muscles of the UL [i.e., mechanical stimulation of the muscle area, using tension and shear pressure at different intensities to improve flexibility and range of motion of the entire limb, (31)]. Mobilization was performed only in muscles that showed increased tone in the hemiparetic UL. High-frequency therapeutic vibration was also used as a sensory treatment. It consisted of the use of a mechanical device that administers vibrational stimuli of low amplitude, with high frequency (50 Hz), on a focal point in order to target specific muscle and tendon areas; its main objective was to increase sensory input and facilitate muscle contraction (32).

### Outcomes and outcome measures

The outcomes and assessment instruments were selected according to the domains of the International Classification of Functioning, Disability, and Health (ICF) of body function, activity, and participation.

As a primary outcome measure, independence to perform ADL and participation were assessed through the motor activity log (MAL), the functional independence measure (FIM), the box and blocks test (BBT), and the Jebsen hand function test (JHFT).

For the secondary outcome, sensory and motor functions were assessed using the Fugl-Meyer Assessment of the upper extremity. The degree of motor imagery and number of exoskeleton activations by imagination recorded by the Neurobots^®^ software were also evaluated for the experimental group.

#### Motor activity log (MAL)

The MAL test was administered to measure the real-world amount of UL function in the most affected arm (33, 34). The MAL comprises 30 items on two ordinal Likert-type scales related to the amount of use (MAL-AOM) and quality of movement (MAL-QOM). Each item is scored from 0 to 5, where a lower score indicates worse performance. A total score is obtained by computing the average of each scale; the higher the average, the better the quantity and quality of use of the UL (33). On both scales, the minimum clinically important difference (MCID) is 1.0 points if the paretic limb is non-dominant or 1.1 points if the paretic limb is dominant. The patient self-reported UL dominance (35).

#### Functional independence measure (FIM)

The FIM was used to measure functional independence during daily activities by evaluating: personal care, toilet training, mobility, locomotion, communication, and social knowledge (36, 37). In the present study, the FIM was administered through interviews. Each item was scored from 1 to 7, according to the patient's need for assistance. The FIM reported an MCID of 22 points (38).

#### Box and blocks test (BBT)

The BBT measures a patient's manual dexterity. Participants were instructed to transport as many blocks as possible from one compartment to another within 60-s. The individual's score equals the number of blocks transported in 60-s. Higher scores indicate better patient's manual dexterity. The test was performed bilaterally to verify that the participant understood the instructions; however, only the score of the paretic hand was considered (39–41). The MCID is 5.5 blocks per minute (42). Individuals who did not transport any blocks within the required time were not considered for the analysis of this outcome.

#### Jebsen hand function test (JHFT)

The JHFT consists of six tasks: turning cards, turning common small objects, simulating feeding, stacking chips, moving large lights, and moving heavy objects (43, 44). Each task was timed, and the patient had a maximum of 120 s to perform each task. Longer times indicate worse performance. Participants who did not perform each task within the given time were not considered for the analysis of results for this evaluation.

#### Fugl-Meyer assessment (FMA)

The FMA for the UL was administered to evaluate sensorimotor impairment. The FMA comprises two domains: motor and sensory. Each item consists of a three-point ordinal scale (0-not able to perform, 1-performs partially, 2-performs fully), with a total score of 66 and 12 points for the motor and sensory domains, respectively (45, 46). The MCID is 5.25 points for the motor domain and 1.2 points for the sensory domain (47).

#### Degree of motor imagery and amount of exoskeleton activations

The Neurobots^®^ software recorded the neurophysiological signals, through non-invasive electrical sensors, during the mental practice (movement imagination) and transformed them into a degree of motor imagery. If the measured degree of motor imagery was maintained above the minimum threshold of 70 points (i.e., amount of desynchronization calculated by the software algorithm) and remained above 70 points for a minimum of 3 s, it would lead to the movement of an exoskeleton that was attached to the patient's hand (for details, refer to “Brain-computer interface” section). At the end of each exercise and session, the software computed an average of the degree of motor imagery reached during the mental practice, the amount of exoskeleton activations, and a heat map indicating which area of the cerebral cortex was more activated during the exercise session. The representation of the image with darker/warmer colors indicated greater brain activation. It was expected that an increase in the degree of motor imagery would also increase the number of exoskeleton activations.

### Data analysis

For the study, we performed distribution analysis using the Shapiro-Wilk test. In order to analyze differences in baseline characteristics between groups, independent sample t-test (normal data distribution) or Mann–Whitney *U*-test (non-normal distribution) was used while for categorical variables, Fisher's exact test was applied. Given the non-normal distribution of post-intervention data of primary outcomes, non-parametric statistics (Mann–Whitney *U*-test) were used between baseline and post-intervention, and between-group comparisons were evaluated using the Wilcoxon and Mann–Whitney sign tests, respectively. The results were interpreted according to Cohen (48) as trivial for *d* < 0.20, small for 0.20 ≤ *d* < 0.50, moderate for 0.50 ≤ *d* < 0.80, and large for *d* ≥ 0.80. The chi-square (χ^2^) test and odds ratio were used to compare differences between groups in the proportion of participants who achieved minimum clinically important difference (MCID) values. The MCID was descriptively analyzed to understand which therapy was superior to help the participant achieve the minimum expected difference for each outcome measure. The sample size was determined based on the expected effect size (*dz*) = 0.7, for within-group analysis. Thus, for α = 0.05 and β = 80%, a sample size of *n* = 19 subjects (+15% accounting for possible dropouts) per group was estimated (*t*-tests–matched pairs, G^*^power) (49). The Statistical Package for the Social Sciences (SPSS) version 22 software was used for all statistical analyses, adopting a significance level of *p* ≤ 0.05.

## Results

**Figure 2:**
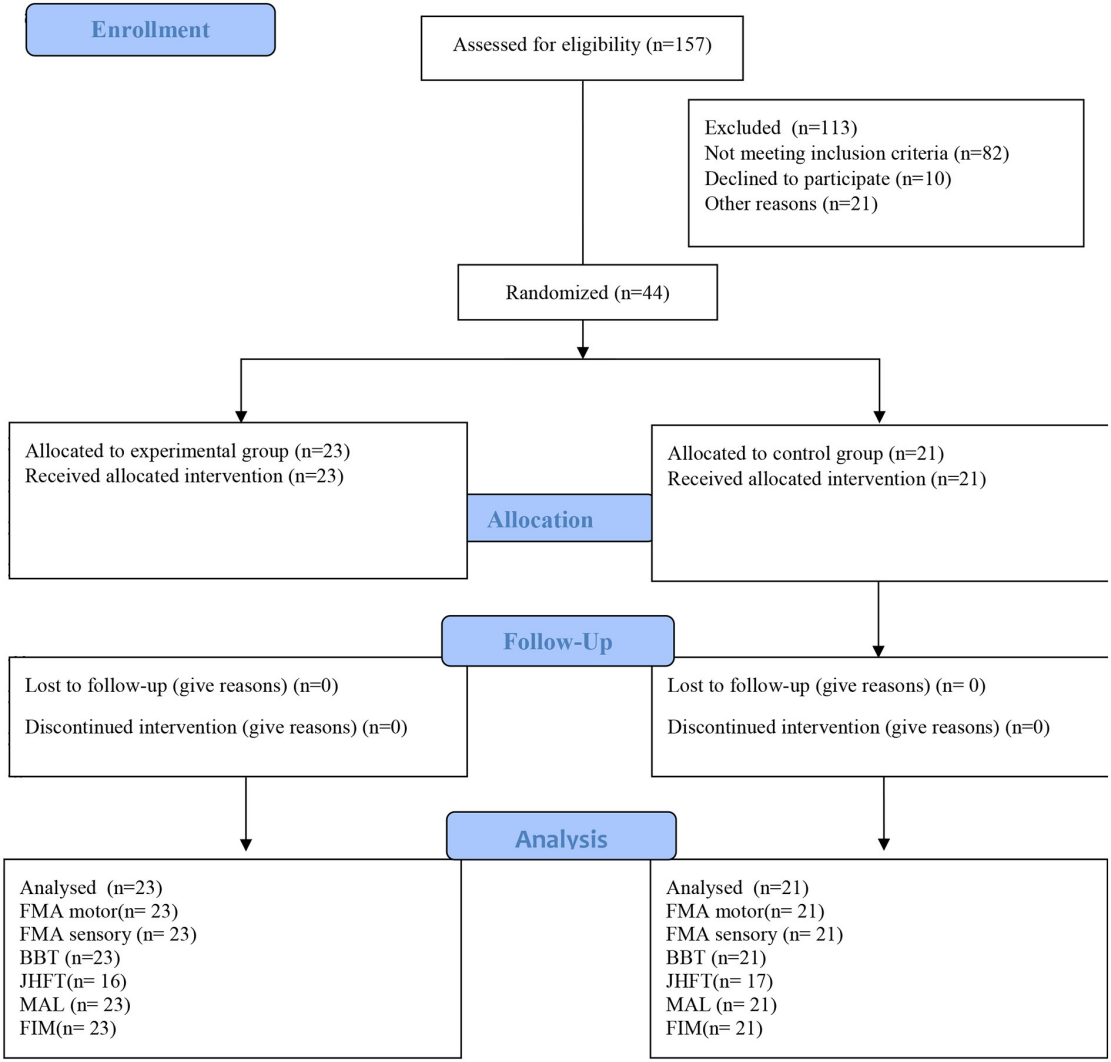
CONSORT flow diagram. FMA, motor, upper limb Fugl-Meyer for motor function; FMA, sensory, upper limb Fugl-Meyer assessment for sensory function; BBT, box and block test; FIM, functional independence measure; JHFT, Jebsen hand function test; MAL, motor activity long test.

Figure 2 shows the flow describing the participants in each study phase. There were no demographic and clinical differences between groups at the baseline (Table 1). No adverse events were reported. Seven participants in the experimental group and four in the control group were unable to perform the JHFT.

**Table 1:** Demographic and stroke characteristics for each group at baseline.

	**Experimental (*n* = 23)**	**Control (*n* = 21)**	***p*** **-value**
Age	62.2 ± 9.8	61 ± 3	0.769^b^
Gender (male)	12 (52.2%)	11 (52.4%)	0.989^a^
Dominance (right)	21 (91.3%)	19 (90.5%)	0.924^a^
Hemiparesis (right)	10 (43.5%)	11 (52.4%)	0.555^a^
Stroke time (weeks)	13.9 ± 6	12.5 ± 6.7	0.388^b^
Ischemic stroke	21 (91.3%)	17 (80.9%)	0.318^a^
MMSE	23.6 ± 4	21.8 ± 3.8	0.107^b^
FMA-motor	32.1 ± 16.8	37 ± 13.5	0.410^b^
FMA-sensory	6.6 ± 2.4	7.7 ± 2.5	0.191^b^
MAL-AOM	0.5 ± 0.6	0.4 ± 0.3	0.981^b^
MAL-QOM	0.6 ± 0.9	0.7 ± 0.6	0.202^b^
BBT	12.2 ± 12.6	11.1 ± 9.8	0.915^b^
JHFT	254.6 ± 239.8	222 ± 171	0.122^b^
FIM	97.6 ± 22.2	99.4 ± 20.4	0.823^b^

Data are mean ± standard deviation and percentages; MMSE, mini mental state examination; FMA, Fugl-Meyer Assessment; MAL, motor activity log; AOM, quantitative; QOM, qualitative; BBT, box and blocks test; JHFT, Jebsen hand function test; FIM, functional independence measure.

a Chi-square test.

b Mann–Whitney *U*-test.

There was a difference between baseline and post-intervention analysis for the experimental group in all assessments (FIM, MAL—AOM and QOM, BBT, and JHFT). There was a statistical difference for the control group only for the MAL and BBT. Although both groups showed differences for the MAL and BBT, the effect size was greater for the experimental group (Table 2). In the between-group analysis, post vs. post, no differences were found for MAL-QOM (Mann–Whitney *U*-test, *p* = 1.00), MAL-AOM (Mann–Whitney *U*-test, *p* = 1.00), BBT (Mann–Whitney *U*-test, *p* = 0.396), FIM (Mann–Whitney *U*-test, *p* = 0.128), and JHFT (Mann–Whitney *U*-test, *p* = 0.65).

**Table 2:** Result of the primary outcome performance in activities of daily living and participation assessed by the functional independence measure, motor activity log, box and blocks test, and Jebsen hand functional test.

	**Experimental**				**Control**			
	**Baseline**	**Post**	***p***	**Cohen's** ***d***	**Baseline**	**Post**	***p***	**Cohen's** ***d***
FIM	97.6 ± 22.2	109.6 ± 20.4^*^	0.001	0.56	99.4 ± 20.4	105.3 ± 19.4	0.072	0.29
MAL-AOM	0.5 ± 0.6	1.4 ± 1.3^*^	0.001	0.83	0.4 ± 0.3	0.7 ± 0.5^*^	0.001	0.72
MAL-QOM	0.6 ± 0.9	1.7 ± 1.6^*^	0.006	0.84	0.7 ± 0.6	1 ± 0.6^*^	0.013	0.50
BBT	12.2 ± 12.6	17.9 ± 15.3^*^	0.004	0.40	11.1 ± 9.8	13.5 ± 11.2^*^	0.005	0.23
JHFT	254.6 ± 239.8	159 ± 176.3^*^	0.001	0.45	222 ± 171	232.8 ± 210.1	0.683	0.05

Cohen's *d*: trivial for *d* < 0.20, small for 0.20 ≤ d < 0.50, moderate for 0.50 ≤ *d* < 0.80, and large for *d* ≥ 0.80. FIM, functional independence measure; MAL, motor activity log; AOM, amount of movement; QOM, quality of movement; BBT, box and blocks test; JHFT, Jebsen hand functional test.

* *p* ≤ 0.05.

### Secondary outcome

**Figure 3:**
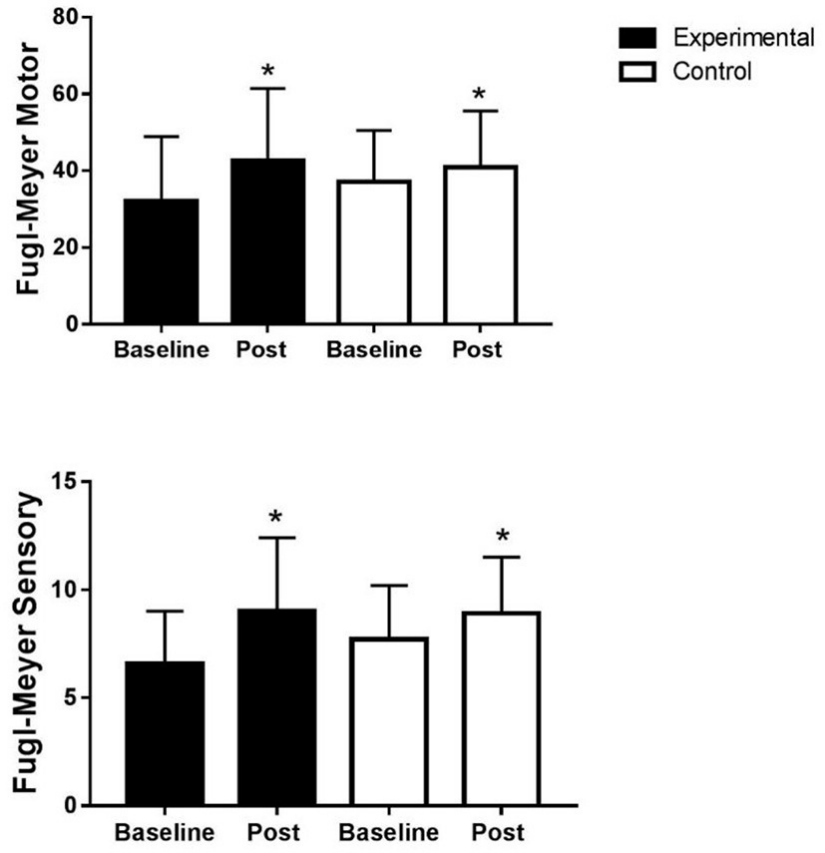
Motor and sensory function of the upper limb assessed by Fugl-Meyer. Fugl-Meyer experimental motor domain (32.1 ± 16.8 vs. 42.6 ± 18.8), sensory domain (6.6 ± 2.4 vs. 9 ± 3.4), control motor domain (37 ± 13.6 vs. 40.9 ± 14.7), and sensory domain (7.7 ± 2.5 vs. 8.9 ± 2.6). *Wilcoxon test, *p* ≤ 0.05.

There was a statistical difference in the FMA for the experimental group in the motor (*p* = 0.001, Cohen's *d* = 0.6) and sensory function (*p* = 0.001, Cohen's *d* = 0.8) as well as for the control group in the motor (*p* = 0.005, Cohen's *d* = 0.3) and sensory function (*p* = 0.012, Cohen's *d* = 0.5). Notably, the effect size was larger in the experimental group (Figure 3). At post-treatment, there was no difference between groups for the FMA motor domain (Mann–Whitney *U*-test, *p* = 0.224) and sensory domain (Mann–Whitney *U*-test, *p* = 1.00).

**Figure 4:**
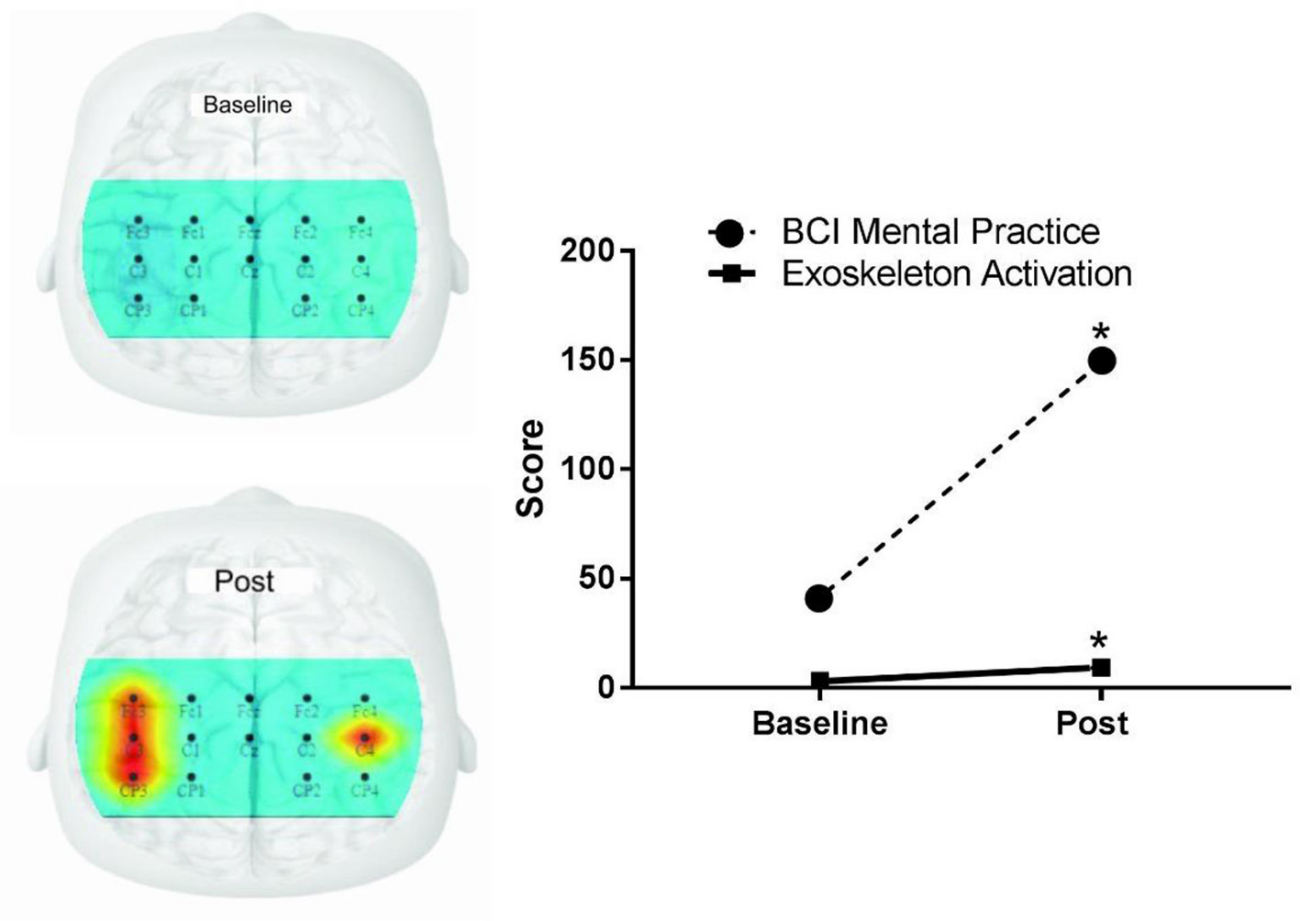
Activation of the premotor cortex, primary motor, primary somatosensory brain areas, degree of motor imaginary, and exoskeleton activations through imagination. BCI, brain-computer interface. The image represents the target brain areas of the premotor cortex, primary motor, and primary somatosensory, measured using the 10-10 electroencephalogram system. The hot color indicates greater activation in both cerebral hemispheres. Skeletal activation indicates how many times the orthosis has been moved through thought.

There was a statistical difference from baseline to post in the experimental group for the degree of motor imaginary (*p* < 0.001) and the amount of exoskeleton activations during the BCI treatment (*p* < 0.001) (refer to Figure 4).

### Minimum clinically important difference (MCID)

**Figure 5:**
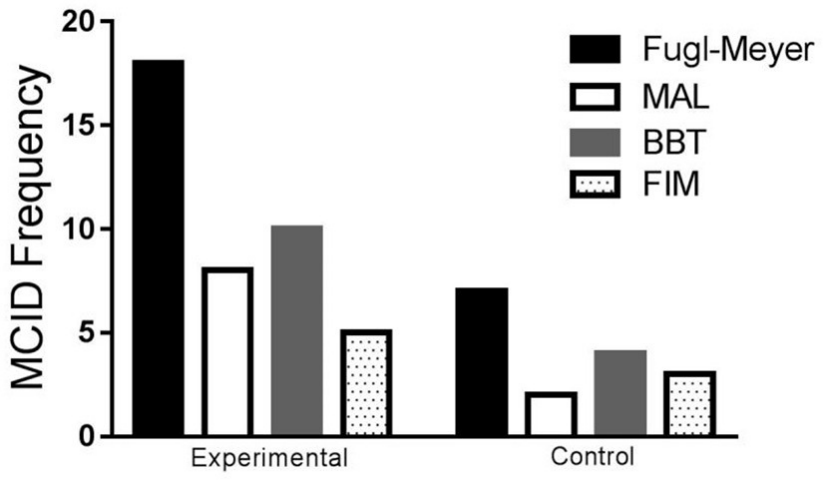
Frequency of participants that achieved the minimum clinically important difference in both groups for the Fugl-Meyer, MAL, BBT, and FIM. MCID, minimal clinically important difference; MAL, motor activity log (MCID 1.0); Fugl-Meyer (MCID 5.25); BBT, box and blocks test (MCID 5.5/min); FIM, functional independence measure (MCID 22).

The proportion of patients who achieved MCID is depicted in Figure 5. Table 3 shows the differences between pre- and post-intervention within each group. Statistical differences were found in the percentage of participants that achieved the MCID in the experimental group compared to the control group for MAL-AOM [$\chi_{\left(1,\text{n}=44\right)}^{2}$ = 3.988, *p* = 0.046; odds ratio = 5.067, 95% CI (0.934, 27.484)], MAL-QOM [$\chi_{\left(1,\text{n}=44\right)}^{2}$ = 6.080, *p* = 0.014; odds ratio = 10.667, 95% CI (1.201, 94.738)], and FMA motor domain [$\chi_{\left(1,\text{n}=44\right)}^{2}$ = 9.031, *p* = 0.003: odds ratio = 7.200, 95% CI (1.879, 27.592)]. No statistical differences were detected for BBT, FMA sensory function, and FIM.

**Table 3:** Delta and minimal clinically important difference.

	**Experimental**	**Control**	**MCID**
	**Delta**	**Delta**	
FIM	12 ± 1.8	5.9 ± 1	22
MAL-AOM	0.9 ± 0.7	0.3 ± 0.2	1.0
MAL-QOM	1.1 ± 0.7	0.3 ± 0	1.0
BBT	5.7 ± 2.7	2.4 ± 1.4	5.5
JHFT	95.6 ± 63.5	10.8 ± 39.1	–

FIM, functional independence measure; MAL, motor activity log; AOM, amount of movement; QOM, quality of movement; BBT, box and blocks test; JHFT: Jebsen hand functional test. Delta represents differences between pre- and post-intervention within each group. MCID represents the cut-off values; for more details, refer to the “Methods” section.

## Discussion

Several neurorehabilitation interventions have investigated UL sensorimotor recovery ADL and participation in post-stroke individuals [e.g., (50, 51)]. While most clinical trials were developed on specific neurorehabilitation interventions, less attention was given to motor control (52) combined with OT and quality of movement (53, 54). The present study aimed to investigate the effects of BCI combined with MP and occupational therapy (OT) on the manual function to improve performance in executing ADL and increase the social participation of stroke survivors in the subacute phase.

The current study showed significant improvements for the experimental group in all outcomes after the BCI combined with MP and OT. Notably, following BCI intervention, the experimental group showed improvements in the degree of motor imagery and the amount of exoskeleton activations. In the control group, post-intervention differences were detected for MAL-AOM, MAL-QOM, and BBT. While no differences were found between groups at post-intervention, the experimental group showed larger effect sizes than those in the control group. Overall, the present study finding highlighted the positive impact of BCI combined with MP and OT on UL sensorimotor recovery, ADL performance improvement, and participation in subacute post-stroke individuals.

Brain-computer interface is a promising strategy for treating and recovering functions, specifically UL motor skills (55). A meta-analytic study with 235 subjects suggested that BCI may be an effective intervention for post-stroke UL motor rehabilitation (6).

In the same sense, it is clinically known that improvement in motor skills does not always mean improvement in functional independence and performance in ADL. Few studies using the BCI clearly showed functional motor responses in the UL (18, 55, 56). However, the question remains whether UL motor improvements can favor performance in ADL.

In order to clarify this scientific gap, this study aimed to investigate the effects of BCI combined with MP and occupational therapy (OT) on the manual function to improve performance in executing ADL and increase the social participation of stroke survivors in the subacute phase.

The experimental group showed a statistically significant improvement in all activity and participation assessments. This fact explains why BCI can provide an additional opportunity to engage in ADL, taking advantage of a good window of plastic recovery of the central nervous system in a shorter time, increased cortical excitability, and reorganization of the neural network of the injured hemisphere, as well as, allowing relearning of movement patterns more similar to what was expected in daily activities with a positive impact on performance in real-world ADL (57, 58).

The improvement in functional independence and the marked increase in activity/participation in the experimental group are explained by the fact that during the BCI protocol, functional tasks were trained using mental practice and later in the effective training of these same functional tasks. In addition, mental practice with functional tasks improved the quality of movement and performance in ADL on the affected side (56, 59).

In the same sense, teaching through observation, imagination, and execution with parameters of the best forms and movement patterns to perform an activity during mental practice with functional tasks has also influenced the quantity and quality of the use of the UL in ADL, evaluated by the motor activity log.

An interesting result was that the control group showed a significant response in two of the four scales for the primary outcome (MAL—qualitative and quantitative and in the BBT), with the effect size for the experimental group being larger. Similar results were observed in a randomized clinical trial using BCI in combination with other therapies, where both groups showed functional improvement after the interventions (55). However, the functional gains obtained on standardized scales were greater in the experimental group, demonstrating the positive role of BCI in post-stroke rehabilitation.

The combined use of BCI with rehabilitation interventions such as physical therapy and occupational therapy appears promising for treating functional problems. Previous studies have found similar results when using BCI associated with conventional therapy, especially in improving motor function (11, 60, 61). Thus, the association between BCI, mental practice, and occupational therapy may have enhanced the reorganization of cortical function, offered centrally and peripherally, improving motor control more than in patients undergoing isolated therapies.

Previous studies suggest [reviewed in (62)] that combined multimodal therapies, similar to those used in our study, probably enhance the effectiveness of each technique, resulting in individual effects on cortical excitability while simultaneously improving sensory-motor processing. Multimodal therapies may partially explain the improvement observed in the experimental group. This approach may be more successful in promoting post-stroke functional recovery by allowing simultaneous access to the injured cortical network at the central and peripheral levels.

Finally, combining peripheral rehabilitation therapies with therapies that directly promote cortical excitability can help to lengthen the therapeutic window, thus offering a greater opportunity for physical and occupational therapies to promote recovery in everyday activities (63).

Both groups showed a statistically significant improvement for the secondary outcome, sensory and motor function, assessed by FMA. A likely explanation is that the groups received conventional therapy with a specific OT protocol. In parallel, the control group improved sensorimotor functions in the same way as the experimental group, probably because they were in the subacute stage of stroke. Bernhardt et al. (22) showed that in the subacute stage of stroke, there is a decrease in neuroinflammation and an increase in spontaneous cortical reorganization. Finally, this spontaneous reorganization is often associated with limited restoration of function, and the rehabilitation process is essential for directing and supporting adaptation to avoid the maladaptive plasticity of neural circuits (7, 9).

Promisingly, BCI may be instrumental in opening an instant window into brain activity and mechanisms that support functional recovery, even if brain activation is not in the specific injured area. However, improving synaptic projections and connections can promote overall improvement in the functioning brain. The view is that BCI not only allows direct control of a robotic device to restore or improve patients' performance but also feeds back into ongoing brain changes related/induced by the BCI-guided exercise itself. Despite the improvement in sensory and motor function in both groups, the experimental group showed larger effect sizes.

Another finding, referring to the secondary outcome, was the increase in the degree of motor imagery and the number of activations of the exoskeleton device during the “Think” phase. The software used captures the neurophysiological signals during the mental practice and transforms them into a degree of motor imagery, which, if kept by the patient within the minimum threshold, leads to activations of the orthosis. Therefore, it is expected that increasing the degree of motor imagery will also result in an increase in the number of activations. This finding about the increase in imagery and device activations can be explained by the learning generated from the repetitions that the BCI provides and the stimulus to mental practice.

The minimum clinically important difference is an important metric, as not every score increase can be translated into clinically relevant improvements. The proportion of patients who achieved MCID was higher in the experimental group compared to the control group. Specifically, concerning the MAL-AOM, MAL-QOM, and FMA motor domains, the fraction of participants receiving BCI combined with MT and OT who reached clinically meaningful improvements was higher than that of the control group. No differences were found in other outcomes. One of the explanations could be that treatment in the experimental group focused more on the upper extremity motor impairments, while gross manual dexterity and functional independence were equally targeted in both arms of the trial. Additionally, it should be noted that the MCID is affected by the initial severity of the impairment, the sample's heterogeneity, and the scale's ordinal nature (64, 65). Thus, the responsiveness along Likert-type scales could be affected (66).

Although no differences were found between the groups, participants in the experimental group showed statistically significant differences at baseline and post-intervention in outcomes that the control group did not achieve. An improvement was expected in the control group because the patients were undergoing an occupational therapy protocol and they were in the subacute phase, in which recovery following rehabilitation is expected (51). Remarkably the experimental group showed improvements in more outcomes than the control group.

This study has some limitations. The sample size (*n* = 44) was small. Nevertheless, the error for type II sample analysis was not influenced. Some subjects (*n* = 11) were unable to perform the JHFT; thus, the effect of the study interventions on fine and gross motor hand functions should be interpreted cautiously. Additionally, even if the outcome measures for assessing UL sensorimotor performance used in the study report satisfactory psychometric properties, laboratory-based assessments such as the kinetics and kinematics of UL could provide more information on the quality of movement. We also consider the large heterogeneity of the sample as a limitation, represented by the large standard deviation in each sample; we suggest that future studies time-stratify stroke onset. Finally, the study design did not control for relevant social participation factors of participants, i.e., work status and retirement. The factors mentioned above should be considered when planning future studies.

## Conclusion

The study showed a positive trend in the clinical effects of the combined use of BCI with mental practice and occupational therapy in the aspects of sensory, motor, and functional independence recovery. BCI is a potential new strategy to improve performance in ADL and social participation in individuals with stroke, specifically in the subacute phase. Future studies are needed to confirm these findings and determine new neurofunctional bases.

## Data availability statement

The raw data supporting the conclusions of this article will be made available by the authors, without undue reservation.

## Ethics statement

The studies involving human participants were reviewed and approved by the Ethics Committee for Human Research at the Federal University of Sergipe. The patients/participants provided their written informed consent to participate in this study.

## Author contributions

AZ: idea conception, text writing, and collected the data. DP: text writing and data analysis. VS: text writing and collected the data. KS, MBa, and LS: text writing. MBo: data analysis and revision process. SS: revision process, results' analysis, editing, language, grammar, and concept revision. KM-S and RD: revision process and results' analysis. All authors contributed to the article and approved the submitted version.
